# BDNF-Overexpressing Engineered Mesenchymal Stem Cells Enhances Their Therapeutic Efficacy against Severe Neonatal Hypoxic Ischemic Brain Injury

**DOI:** 10.3390/ijms222111395

**Published:** 2021-10-22

**Authors:** So Yoon Ahn, Dong Kyung Sung, Yun Sil Chang, Se In Sung, Young Eun Kim, Hyo-Jin Kim, Soon Min Lee, Won Soon Park

**Affiliations:** 1 Department of Pediatrics, Samsung Medical Center, Sungkyunkwan University School of Medicine, Seoul 06351, Korea; soyoon.ahn@samsung.com (S.Y.A.); dbible@naver.com (D.K.S.); yschang@skku.edu (Y.S.C.); sein.sung@samsung.com (S.I.S.); 2 Stem Cell and Regenerative Medicine Institute, Samsung Medical Center, Seoul 06351, Korea; duddms0920@naver.com; 3 SL BiGen, Inc., SL BIGEN Research Hall, 85, Songdogwahak-ro, Yeonsu-gu, Incheon 21983, Korea; hjkim@slbigen.com (H.-J.K.); smlee@slbigen.com (S.M.L.)

**Keywords:** hypoxia-ischemia, brain, cell transplantation, stem cells, mesenchymal stem cell transplantation, infant, newborn, brain derived neurotropic factor

## Abstract

We investigated whether irradiated brain-derived neurotropic factor (BDNF)-overexpressing engineered human mesenchymal stem cells (BDNF-eMSCs) improve paracrine efficiency and, thus, the beneficial potency of naïve MSCs against severe hypoxic ischemic (HI) brain injury in newborn rats. Irradiated BDNF-eMSCs hyper-secreted BDNF > 10 fold and were >5 fold more effective than naïve MSCs in attenuating the oxygen-glucose deprivation-induced increase in cytotoxicity, oxidative stress, and cell death in vitro. Only the irradiated BDNF-eMSCs, but not naïve MSCs, showed significant attenuating effects on severe neonatal HI-induced short-term brain injury scores, long-term progress of brain infarct, increased apoptotic cell death, astrogliosis and inflammatory responses, and impaired negative geotaxis and rotarod tests in vivo. Our data, showing better paracrine potency and the resultant better therapeutic efficacy of the irradiated BDNF-eMSCs, compared to naïve MSCs, suggest that MSCs transfected with the BDNF gene might represent a better, new therapeutic strategy against severe neonatal HI brain injury.

## 1. Introduction

Despite recent improvements in perinatal and neonatal intensive care medicine, neonatal hypoxic ischemic encephalopathy (HIE) remains a major cause of neonatal mortality and long-term neurologic morbidities, such as intellectual disability, cerebral palsy, and epilepsy in survivors [1,2]. While therapeutic hypothermia is the only currently applicable therapeutic option in clinical practice to improve the outcomes of neonatal HIE [3,4], more than half of infants with HIE expire or develop severe neurologic problems, especially in severe HIE [5,6]. Therefore, developing a new treatment in addition to therapeutic hypothermia to improve the outcome of severe neonatal HIE is an urgent issue.

Recently, we and others have reported the neuroprotective therapeutic effects of mesenchymal stem cell (MSC) transplantation in HI brain injury [7,8], stroke [9], and intraventricular hemorrhage (IVH) neonatal rat pup models [10,11]. We also showed that concurrent or delayed MSC injection with hypothermia treatment synergistically improved severe HI brain injury in contrast to hypothermia-alone therapy [12,13]. These findings suggest that MSC transplantation might be a novel clinically effective therapy in addition to therapeutic hypothermia to improve the outcome of currently intractable severe neonatal HIE. However, despite the enthusiasm based on these reports on therapeutic potentials, high variability of MSC preparations, according to donor characteristics, tissue origin, methods of isolation and culture conditions [14,15,16], and their limited life span for ex vivo expansion [17], are major hurdles for the successful translation of MSC transplantation to the clinic. A possible solution to circumvent the heterogeneity and limited growth expansion of MSCs is immortalization by genetic manipulation, thereby producing a new human MSC cell line that preserves their paracrine and differentiating characteristics and enables large-scale production at a much lower costs that is ready for both preclinical and clinical use [18,19,20].

The pleiotropic neuroprotective anti-inflammatory, anti-fibrotic, anti-oxidative, anti-apoptotic, and permeability-decreasing effects of transplanted MSCs are mediated predominantly by their paracrine response via the secretion of various biological factors, including brain-derived neurotropic factor (BDNF) rather than by direct regeneration [21,22,23]. Previously, we observed that BDNF secreted by transplanted MSCs played a critical role in mediating their beneficial effects, such as the attenuation of cell death, inflammation, astrogliosis, and the development of post-hemorrhagic hydrocephalus, and improved neurogenesis and myelination after severe IVH in newborn rats [1]. However, although the BDNF level was significantly increased with MSC transplantation compared to the intraventricular hemorrhage (IVH) control group, the absolute BDNF level was still much lower than that in the normal control group. These findings suggest that MSCs overexpressing BDNF might enhance the neuroprotective effects of MSC transplantation. Kurozumi et al. [24] reported that BDNF-eMSCs produced 23-fold greater BDNF than naïve MSCs, promoted functional recovery, and reduced the number of TUNEL-positive cells and infarct size in a rat transient middle cerebral artery occlusion (MCAO) model. In contrast, van Velthoven et al. [25] reported that intranasal transplantation of BDNF-hypersecreting MSCs after neonatal stroke did not further enhance the recovery of MCAO-induced brain injury with naïve MSCs transplantation. Therefore, the issue of transplanting BDNF-overexpressing MSCs to promote their therapeutic efficacy must be addressed in future studies before clinical use can be considered. In this study, we investigated whether genetically modifying engineered human MSCs to secrete more BDNF enhances the therapeutic efficacy of naïve human MSCs in vitro for oxygen glucose deprivation (OGD) and in vivo in newborn rats received therapeutic hypothermia after severe HI brain injury.

## 2. Results

### 2.1. Cellular Characteristics of BDNF-eMSCs

**Figure 1:**
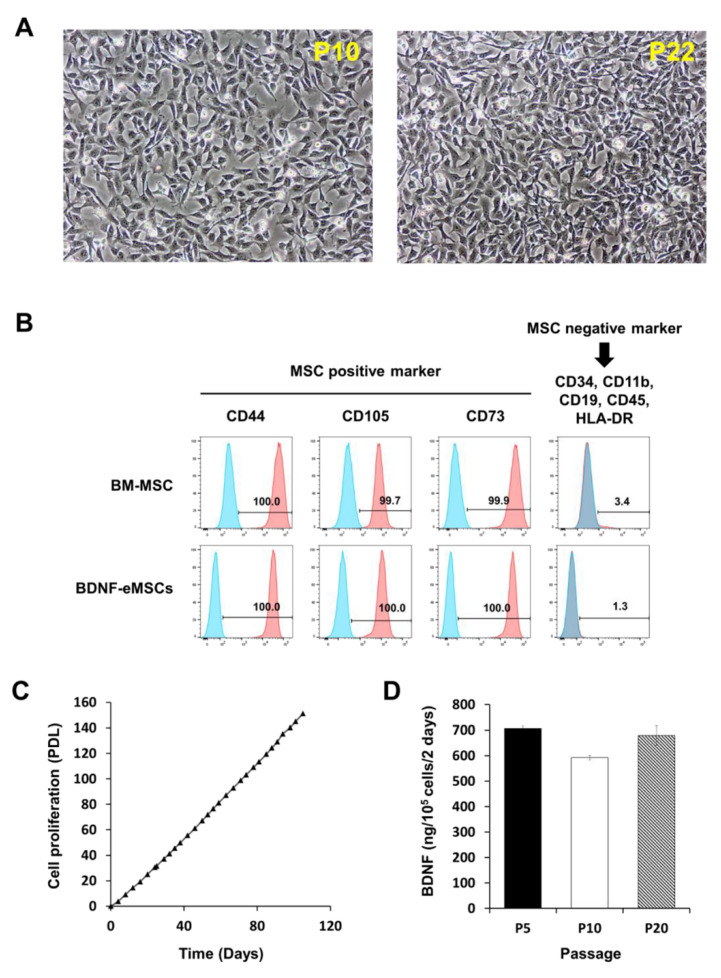
Characterization of BDNF- overexpressing engineered mesenchymal stem cells (eMSCs). (**A**) Morphology of BDNF-eMSCs. (**B**) Flow cytometry analyses show that BDNF-eMSCs express specific markers for MSCs such as CD44, CD105, and CD73. N = three per group (**C**) Cell proliferation rate of BDNF-eMSCs. This result is representative of three independent experiments. (**D**) BDNF secretion of distinct passages of BDNF-eMSCs measured by ELISA.

The genetically engineered BDNF-eMSCs displayed a homogenous spindle-shaped cell morphology, indistinguishable from naïve MSCs (Figure 1A), expressed the same markers specific for MSCs, such as CD44, CD73, CD90, and CD105, without expressing CD34, CD11b, CD19, CD45, and human leukocyte antigen-DR (Figure 1B), demonstrated highly proliferative potential exhibited by linear population doubling levels up to 120 days of culture (Figure 1C), and stably secreted human BDNF protein up to 20 passages (Figure 1D).

### 2.2. Cell Viability, Cytotoxicity, Oxidative Stress, and TUNEL Assay after In Vitro OGD

**Figure 2:**
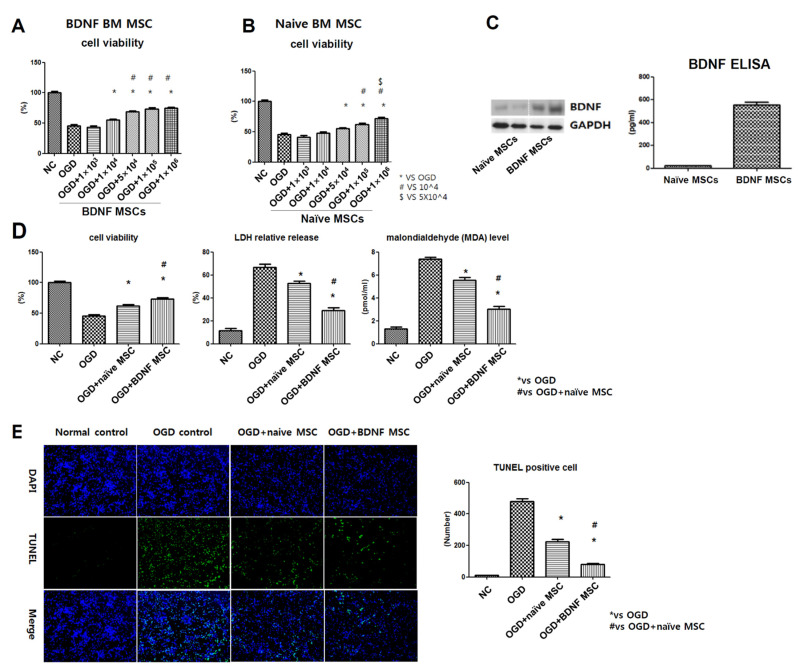
Neuroprotective efficacy of BDNF-eMSCs in vitro oxygen–glucose deprivation (OGD)-induced primary cultured rat cortical neurons. Cell viability, which is expressed as relative proliferation rate (%) to normal control group, was assessed with the CCK-8 assay in OGD-induced cultured neurons after co-treatment with BDNF-eMSCs, at doses of approximately 1 × 10^3^, 1 × 10^4^, 5 × 10^4^, 1 × 10^5^, 1 × 10^6^ cells per 1 mL, (**A**) and with naïve MSCs at doses of approximately 1 × 10^3^, 1 × 10^4^, 5 × 10^4^, 1 × 10^5^, 1 × 10^6^ cells per 1 mL (**B**). (**C**) Western blotting and ELISA assay for BDNF expression from the culture media of naïve MSCs and BDNF-eMSCs. (**D**) Cytotoxicity was evaluated with cell viability, expressed as a relative proliferation rate (%) to the normal control group, relative lactate dehydrogenase (LDH) release (%) to the positive control (100% fully killed cells), malondialdehyde (MDA) level in neuronal cells after OGD induction with/without co-treatment of naïve MSCs or BDNF-eMSCs at a dose of 1 × 10^5^ cells per 1 mL. (**E**) The number of terminal deoxynucleotidyl transferase UTP nick end labeling (TUNEL)-positive cells evaluated in OGD-induced rat cortical neurons after co-culture with naïve MSCs or BDNF-eMSCs at dose of 1 × 10^5^ cells per 1 mL. Data are expressed as the mean ± SD.

In primary cultures of rat cortical neurons in vitro, oxygen glucose deprivation (OGD) was induced. After OGD induction, naïve MSCs or BDNF-eMSCs were co-treated with different doses: approximately at concentrate of 1 × 10^3^, 1 × 10^4^, 5 × 10^4^, 1 × 10^5^, 1 × 10^6^ cells per 1 mL. For the in vitro dose test, naïve MSCs significantly and dose-dependently reduced OGD-induced cell death with doses of 5 × 10^4^, 1 × 10^5^, 1 × 10^6^ cells per 1 mL. Then, BDNF-eMSCs significantly and dose-dependently reduced OGD-induced cell death with doses of 1 × 10^4^, 5 × 10^4^, 1 × 10^5^, 1 × 10^6^ cells per 1 mL. (Figure 2A,B). When compared to naïve MSCs, BDNF-eMSCs seem to secrete BDNF ten times more (Figure 2C). When naïve MSCs or BDNF-eMSCs were co-treated with a dose of 1 × 10^5^ cells per 1 mL in the primary cultures of rat cortical neurons, the BDNF-eMSCs co-treated group showed elevated protective effects against cell death and injury those indicated by LDH, MDA, and TUNEL-positive cells (Figure 2D,E). This high-level secretion of BDNF by BDNF-eMSCs may be related to the upgraded therapeutic effects.

### 2.3. Short-Term Follow-Up In Vivo Study of HI-Induced Severe Brain Injury

**Figure 3:**
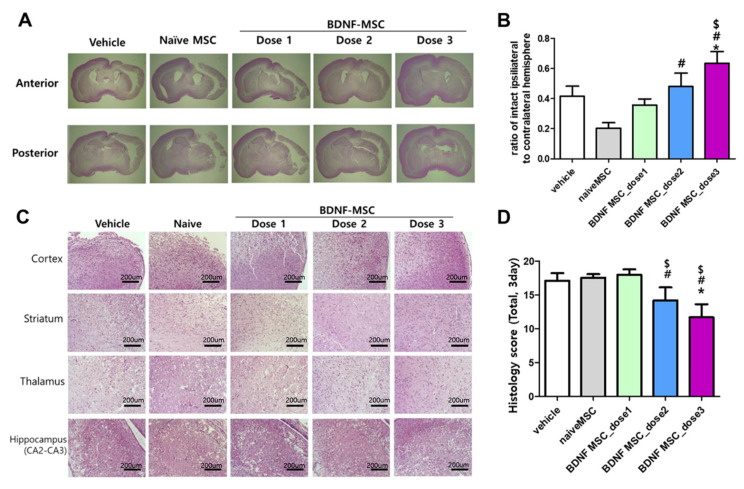
Morphological changes in the brains following hypoxic-ischemic brain injury and MSC transplantation. (**A**) Hematoxylin and eosin (H&E)-stained brain sections showing representative gross morphology of brains at 3 days after modeling. (**B**) The ratio of intact ipsilateral hemisphere to contralateral hemisphere was evaluated in the histologic brain section at 3 days after modeling in each group. (**C**) H&E-stained brain sections showing representative brain histology in cortex, striatum, thalamus, and hippocampus at 3 days after modeling. (**D**) Total sum of histologic scores in each area of brain; cortex, striatum, thalamus, and hippocampus. Data are expressed as mean ± standard error of the mean. * *p* < 0.05 compared to the HI + vehicle control, # *p* < 0.05 compared to the HI + naïve MSCs, $ *p* < 0.05 compared to HI-+ BDNF-eMSCs at dose 1 (approximately 1 × 10^4^ cells). Abbreviations: vehicle, HI + vehicle control, naïve MSCs; HI + naïve MSCs, BDNF MSC_dose1; HI + BDNF-eMSCs at 1 × 10^4^ cells dose; BDNF MSC_dose2; HI + BDNF-MSCs at 5 × 10^4^ cells dose; BDNF MSC_dose3; HI + BDNF-MSCs at 1 × 10^5^ cells dose.

Figure 3A,C show representative light microscopic findings of H&E-stained brain in each study group at 3 days after HI induction for measuring brain infarct and histologic injury scores. HI-induced severe brain infarct and histological injury scores were not significantly attenuated in naïve MSCs (approximately 1 × 10^5^ cells) and dose 1 (approximately 1 × 10^4^ cells) BDNF-eMSCs transplantation, and showed a tendency to be attenuated without statistical significance with dose 2 (approximately 5 × 10^4^ cells) BDNF-eMSCs, and significantly attenuated only with dose 3 (approximately 1 × 10^5^ cells) BDNF-eMSCs transplantation (Figure 3B,D). 

**Figure 4:**
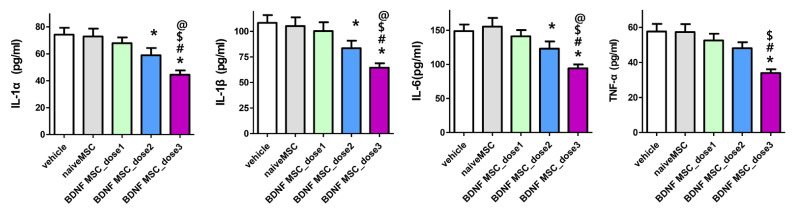
Brain inflammatory cytokines after hypoxic ischemic injury and MSC transplantation. Levels of inflammatory cytokines including interleukin (IL)-1α, IL-β, and IL-6, and tumor necrosis factor (TNF)-α in total brain tissue homogenates at 3 days after modeling. Data are expressed as mean ± standard error of the mean. * *p* < 0.05 compared to the HI + vehicle control, # *p* < 0.05 compared to the HI + naïve MSCs, $ *p* < 0.05 compared to HI + BDNF-eMSCs at dose 1 (approximately 1 × 10^4^ cells), @ *p* < 0.05 compared to HI + BDNF-eMSCs at dose 2 (approximately 5 × 10^4^ cells). Abbreviations: vehicle, HI + vehicle control, naïve MSCs; HI + naïve MSCs, BDNF MSC_dose1; HI + BDNF-eMSCs at 1 × 10^4^ cells dose; BDNF MSC_dose2; HI + BDNF-eMSCs at 5 × 10^4^ cells dose; BDNF MSC_dose3; HI + BDNF-eMSCs at 1 × 10^5^ cells dose.

Increase in the levels of inflammatory cytokines, such as IL-1α, IL-1β, IL-6, and TNF-α, 3 days after severe HI brain injury induction measured at the penumbral area of the brain were not significantly attenuated by naïve MSCs (approximately 1 × 10^5^ cells) and dose 1 (approximately 1 × 10^4^ cells) BDNF-eMSCs transplantation. However, the levels of IL-1α, IL-1β, and IL-6, but not TNF-α, were significantly attenuated with dose 2 (approximately 5 × 10^4^ cells) BDNF-eMSCs, and the attenuation was best observed with dose 3 (approximately 1 × 10^5^ cells) BDNF-eMSCs transplantation (Figure 4).

### 2.4. Long-Term Follow-Up In Vivo Study of Severe HI Induced Brain Injury

**Figure 5:**
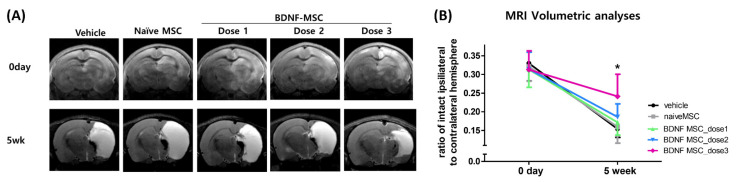
Brain damage area after hypoxic ischemic injury and MSC transplantation. (**A**) Representative serial brain magnetic resonance imaging (MRIs) from each group at right after HI brain injury modeling and 5 weeks after modeling. (**B**) The volumetric ratio of intact ipsilateral hemisphere to contralateral hemisphere was volumetrically evaluated in the serial images of brain MRI in each group right after HI brain injury modeling and 5 weeks after modeling. Data are expressed as mean ± standard error of the mean. * *p* < 0.05 compared to the HI + vehicle control. Abbreviations: vehicle, HI + vehicle control, naïve MSCs; HI + naïve MSCs, BDNF MSC_dose1; HI + BDNF-eMSCs at 1 × 10^4^ cells dose; BDNF MSC_dose2; HI + BDNF-eMSCs at 5 × 10^4^ cells dose; BDNF MSC_dose3; HI + BDNF-eMSCs at 1 × 10^5^ cells dose.

Figure 5A shows representative brain MRI findings of each study group. While the extent of severe brain infarct measured on the day of HI induction was similar between the study groups, the progress of brain infarct measured at 5 weeks after HI induction was significantly attenuated only with dose 3 (approximately 1 × 10^5^ cells), but not with dose 2 (approximately 5 × 10^4^ cells) or dose 1 (1 × 10^4^ cells) BDNF-eMSCs or naïve MSCs (1 × 10^5^ cells) (Figure 5B).

**Figure 6:**
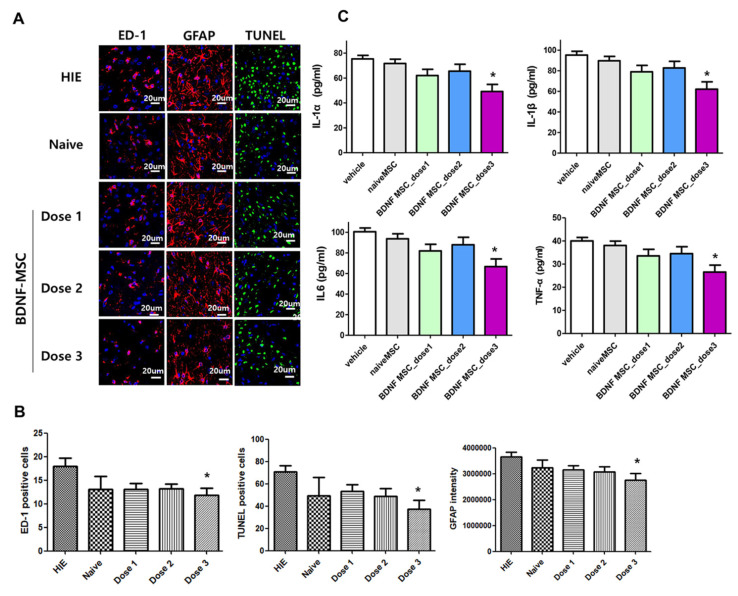
Brain tissue injury including inflammation, cell death, and reactive gliosis after hypoxic ischemic injury and MSC transplantation. (**A**) Representative immunofluorescence photomicrographs of the penumbra area with staining for ED1 (red), glial fibrillary acidic protein (GFAP) (red), TUNEL (green), and 4′,6-diamidino-2-pheylindole (DAPI) (blue) in the brains at 5 weeks after modeling. (**B**) The average number of ED1-positive cells, TUNEL-positive cells, and mean light intensity of GFAP, DCX, and MBP immunofluorescence per high-power field (HPF) in each group. (**C**) Levels of inflammatory cytokines including IL-1α, IL-β, and IL-6 and TNF-α in total brain tissue homogenates at 5 weeks after modeling. Data are expressed as mean ± standard error of the mean. * *p* < 0.05 compared to the HI + vehicle control. Abbreviations: vehicle, HI + vehicle control, naïve MSCs; HI + naïve MSCs, BDNF MSC_dose1; HI + BDNF-eMSCs at 1 × 10^4^ cells dose; BDNF MSC_dose2; HI + BDNF-eMSCs at 5 × 10^4^ cells dose; BDNF MSC_dose3; HI + BDNF-eMSCs at 1 × 10^5^ cells dose.

HI induced an increase in the populations of ED-1-positive cells, GFAP-positive astrogliosis, and TUNEL-positive cells, and increased the levels of inflammatory cytokines such as IL-1α, IL-1β, IL-6, and TNF-α measured in the peri-infarct brain at 5 weeks after HI induction, which was significantly attenuated only with dose 3 (1 × 10^5^ cells), but not with dose 2 (5 × 10^4^ cells) or dose 1 (1 × 10^4^ cells) BDNF-eMSCs or naïve MSCs (1 × 10^5^ cells) (Figure 6).

**Figure 7:**
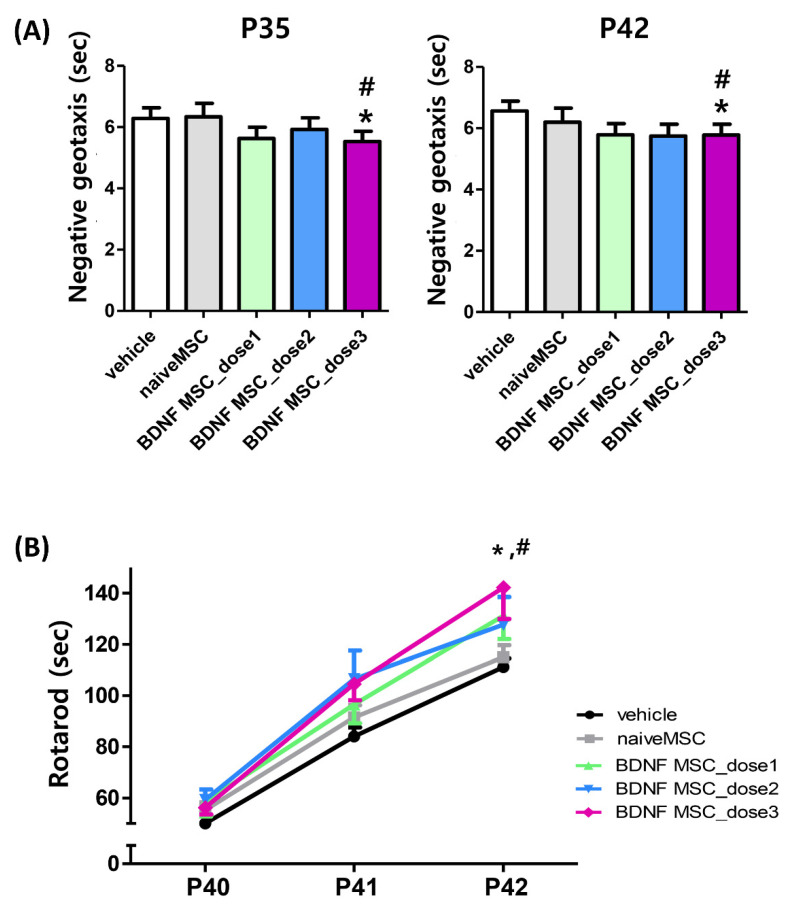
Sensorimotor behavior function after hypoxic ischemic injury and MSC transplantation. Sensorimotor functional outcomes on the negative geotaxis test at P35 (4 weeks after modeling) and at P42 (5 weeks after modeling) (**A**) and rotarod test (**B**) on P40–42 in each group. Data are expressed as mean ± standard error of the mean. * *p* < 0.05 compared to the HI + vehicle control, # *p* < 0.05 compared to the HI + naïve MSCs. Abbreviations: vehicle, HI + vehicle control, naïve MSCs; HI + naïve MSCs, BDNF MSC_dose1; HI + BDNF-eMSCs at 1 × 10^4^ cells dose; BDNF MSC_dose2; HI + BDNF-eMSCs at 5 × 10^4^ cells dose; BDNF MSC_dose3.

Functional behavior tests such as the negative geotaxis test and the rotarod test were performed weekly until five weeks after HI brain injury induction, and for three consecutive days before the end of the 5-week experiment, respectively, on P40, 41, and 42. The HIE-induced prolongation of the negative geotaxis test measured at P35 and P42 (4 and 5 weeks after HI induction) was significantly attenuated only with dose 3 (1 × 10^5^ cells), but not with dose 2 (5 × 10^4^ cells) or dose 1 (1 × 10^4^ cells) BDNF-eMSCs or naïve MSCs (1 × 10^5^ cells) (Figure 7A). While the initial rotarod test results performed at P40 were not significantly different between the study groups, the HI control group displayed an appropriate learning curve with a longer latency to fall at P41 and P42. The longer latency to fall observed in the HI control group was significantly prolonged only with dose 3 (1 × 10^5^ cells), but not with dose 2 (5 × 10^4^ cells) or dose 1 (1 × 10^4^ cells) BDNF-eMSCs or naïve MSCs (1 × 10^5^ cells) transplantation (Figure 7B). 

## 3. Discussion 

Neonatal HIE remains a serious disease, exhibiting high mortality and morbidities in survivors, especially in those with the severe type of HIE, despite clinically available hypothermia treatment [5,6]. The development of an appropriate animal model that stimulates the clinical course of severe neonatal HIE would thus be an essential first step for further delineating its pathophysiologic mechanism and proving the beneficial effects of any novel treatment. In this present study, well-established, relatively inexpensive, and easily mastered Rice–Vannucci model of HI brain injury was used [26]. To overcome the drawback of the broad variability in the severity of HI brain damage and the ensuing brain infarct, which would limit direct comparison between the experiments of this model [27], we randomly assigned the newborn rats into each study group only after confirming the development of severe brain injury involving >50% of the ipsilateral hemisphere volume, as confirmed by brain MRI, conducted within 2 h after the HI insult in this study. Our data, showing the progression of severe HI brain injury to brain infarct via in vivo brain MRI, pathological and biochemical abnormalities, and impaired behavioral function tests in the HI control group, despite hypothermia treatment, showed that this neonatal rat animal model is most suitable for testing the therapeutic effects of MSC transplantation against severe neonatal HI brain injury [12,13]. 

In the present study, we first immortalized naïve MSCs to circumvent the major drawbacks of MSCs, such as their heterogeneity and limited life span for ex vivo expansion by genetic manipulation [14,15,16,17]. Since BDNF is known to be a critical factor for neuronal survival in various disorders including focal ischemia, hypoglycemia, meningitis, and IVH [11,24,28,29,30], we transfected the human BDNF gene to generate BDNF-eMSCs, preserving their phenotypic, paracrine, and differentiating characteristics, and enabling large-scale production at a much lower cost [18,19,20,31]. However, in addition to therapeutic efficacy, safety is another critical issue for successful clinical translation. As genetically engineered BDNF-eMSCs were generated using a lentiviral vector encoding the c-Myc reprogramming factor, tumorigenicity is a major concern for their clinical translation. In this study, after gamma irradiation of the genetically engineered BDNF-eMSCs, we observed the abrogation of MSC proliferation without affecting cell viability and BDNF secretion, and no tumor was detected from in vivo tumorigenicity testing using nude mice. Overall, these findings suggest that the irradiated genetically engineered BDNF-eMSCs are safe and ready to use and their therapeutic efficacy can be tested both in preclinical and clinical studies for treating severe neonatal HI brain injury [31,32,33]. 

In this study, while BDNF secretion in primary cultures of rat cortical neurons in vitro increased >10 fold, the irradiated BDNF-eMSCs were >5 fold more effective than naïve MSCs in attenuating the OGD-induced increase in cytotoxicity, oxidative stress, and cell death. Moreover, in the in vivo newborn rat pup model, severe neonatal HI induced short-term brain injury scores, long-term progression to brain infarct, increased apoptotic cell death, astrogliosis and inflammatory responses, and impaired negative geotaxis, and rotarod tests were significantly attenuated only with irradiated BDNF-eMSCs (approximately 1 × 10^5^ cells), but not with the same dose (approximately 1 × 10^5^ cells) of naïve MSCs transplantation. These findings suggest that the better therapeutic efficacy of irradiated BDNF-eMSCs transplantation against severe neonatal HI-induced brain injuries might be primarily mediated by their enhanced secretion of BDNF. Previously, we observed that BDNF secreted by transplanted MSCs stimulated the intracellular BDNF-TrkB-Akt/Erk-CREB signaling pathway, thereby attenuating neuronal loss and promoting neurogenesis in the hippocampus after severe IVH in newborn rats [34]. Further studies will be necessary to delineate the underlying molecular mechanism of the BDNF secreted by MSCs. While we previously observed the best anti-inflammatory and anti-apoptotic effects, and the resultant best reduction of severe neonatal HI induced brain damage by combined hypothermia and intraventricular administration of approximately 1 × 10^5^ human umbilical cord blood-derived naïve MSCs [12,13], in the present study, delayed intraventricular transplantation of the same treatment failed to significantly induce anti-inflammatory and anti-apoptotic effects, and failed to attenuate the ensuing brain infarction after severe neonatal HI brain injury. These data indicate that the source of MSCs might be a critical factor affecting their secretary capability and thus the beneficial effects of stem cell therapies [35]. Nevertheless, our data on significant neuroprotection along with BDNF hypersecretion against severe neonatal HI brain injury with irradiated BDNF-eMSCs, but not with naïve MSCs transplantation, suggest that BDNF gene modification in MSCs could maximize their secretary capability and the beneficial therapeutic effects regardless of their sources. 

Our data, showing the seminal role of BDNF in mediating neuroprotection against severe neonatal HI brain injury, suggests that the administration of exogenous BDNF might be a novel therapeutic option to improve the outcome of this intractable disorder. However, its short half-life, difficulty in crossing the blood–brain barrier after systemic administration, and lack of large-scale production are major hurdles for its successful clinical translation. Therefore, transplantation of BDNF-eMSCs might be the most effective means of delivering BDNF to the injured brain tissue, and could thus be the most promising therapeutic approach compared to treatment with naïve MSCs or exogenous BDNF alone. In concordance with our data showing better therapeutic efficacy of BDNF-eMSCs than naïve MSCs transplantation against severe neonatal HI brain injury, Kurozumi et al. [24] reported that, along with a 23-fold increase in BDNF production, intracerebral transplantation of BDNF gene-transfected MSCs one day after induction of MCAO promoted functional recovery and reduced infarct size in rats. In contrast, van Velthoven et al. [25] reported that intranasal transplantation of BDNF hypersecreting MSCs at 3 days after MCAO failed to further enhance the MSC-induced recovery of stroke-induced brain injuries in newborn rats. Collectively, these findings suggest that the administration of BDNF gene-modified MSCs is insufficient, and further studies to determine the optimal strategies regarding the route, timing, and dose are warranted for their best therapeutic efficacy against severe neonatal HI brain injury [21].

## 4. Materials and Methods

### 4.1. Cell Preparation

All of the BDNF-eMSCs were provide SL BiGen, Inc., (Incheon, Korea). Primary bone marrow mesenchymal stem cells (naïve MSCs) (Catholic MASTER Cells) were obtained from Catholic Institute of Cell Therapy (CIC, Seoul, Korea). The development and research of this BDNF-eMSC was approved by Public Institutional Review Board Designated by Ministry of Health and Welfare in South Korea (P01-201702-31-003, 08 Feb 2017). Naïve MSCs were cultured in low glucose-containing Dulbecco’s Modified Eagle Medium (DMEM) (Gibco, Grand Island, NY, USA) supplemented with 20% fetal bovine serum (FBS) (Gibco) and 5 ng/mL basic fibroblast growth factor (bFGF) (PeproTech, Rocky Hill, NJ, USA) at 37 °C and 5% CO_2_. In this current study, naïve MSCs of 5th passages were used. 

To generate engineered immortalized MSCs, we first produced a replication-incompetent lentiviral vectors each containing the c-Myc and tetracycline transactivator (tTA) were synthesized (GenScript, Piscataway, NJ, USA) and transfected using the pBD lentiviral vectors (SL BiGen, Inc., Incheon, South Korea) [31]. After infection, survived immortalized MSCs showed high proliferative property and these engineered MSCs expanded and banked to further therapeutic gene transfection.

The reference sequence for BDNF was NP_733927.1. We introduced an optimized DNA sequence into the lentiviral vector. Human BDNF gene containing lentiviral vector transfection to immortalized MSCs. Final monoclonal BDNF-eMSC was selected by BDNF protein secretion, proliferation rate and other MSC phenotypes. BDNF-eMSCs was cultured in low glucose-DMEM supplemented with 10% FBS, 10 ng/mL bFGF and 2 μg/mL doxycycline (Merck, Kenilworth, NJ, USA) at 37 °C at 5% CO_2_. 

All co-treated and implanted BDNF-eMSCs used in in vitro and in vivo efficacy tests were cryopreserved, and then irradiated using high-level gamma irradiation device (MDS Nordion, Ottawa, Ontario, Canada) at KAERI, Advanced Radiation Technology Institute in this study to prevent proliferation [31,32,33]. Because these genetically engineered cells may have some tumorigenic potential, irradiation was carried out only to BDNF-eMSCs to eliminate the possibility of tumorigenicity.

### 4.2. MSC Marker Analysis

Immunophenotype of MSCs was analyzed using flow cytometry. Cells were detached from the flask using trypsin then centrifuged at 1500 rpm for 5 min and re-suspended in staining/wash buffer (PBS with 2% FBS). For staining, naïve MSCs and BDNF-eMSCs were washed with staining/wash buffer and incubated for 30 min in dark at 4 °C with Stemflow^TM^ human MSC Analysis Kit (BD, Franklin Lakes, NJ, USA), according to the manufacturer’s instructions. MSCs were characterized using the following conjugated monoclonal antibody: CD105 PerCP-Cy5.5 (Clone: 266), CD73 APC (Clone: AD2), PE negative cocktail (CD34, CD11b, CD19, CD45, HLA-DR). Flow cytometry was conducted utilizing LSR Fortessa^TM^ cell analyzer (BD) and analyzed by flowJo_V10 or BD FACS Diva software.

### 4.3. Cell Proliferation Analysis

To determine the proliferation rate of BDNF-eMSCs, on average, 6 × 10^5^ cells were seeded into T175 flask (Thermo Fisher Scientific, Waltham, MA, USA). After 3–4 days, cells were harvested and proliferation rate was measured by trypan blue (Gibco) staining method using single-use hemocytometer (INCYTO, Cheonan, Chungnam, Korea). Grown cells were washed, trypsinized, and manually counted on a hemocytometer with trypan blue staining method. Dead or dying cells were stained with trypan blue, we were able to exclude stained cells and count unstained live cells only. Cell population doubling was calculated by measuring the number of cells grown at subculture and by these cumulative data, we were able to calculate the population-doubling level. Cumulative population doubling level (PDL) of BDNF-eMSCs was determined by the summation of population doubling (PD). The PDL was calculated using (logN-logN_0_)/log2, where N = the number of harvested cells and N_0_ = the number of seeded cells.

### 4.4. In Vitro Model of Oxygen–Glucose Deprivation

On day 10 of primary cultured rat cerebral cortical neurons that were isolated on embryonic day 18–19, OGD was carried out to generate cortical neuronal cell death, as described previously [1,36,37]. Briefly, cortical neurons were bubbled with 95% N_2_ and 5% CO_2_ in glucose-free media. The cortical neurons were transferred to an anaerobic chamber (Galaxy 48R incubator; Eppendorf/Galaxy Corporation, Enfield, CT, USA) containing 1% O_2_ and 5% CO_2_ humidified at 37 °C, which was then maintained at a constant pressure of 1500 Pa for 90 min. OGD was terminated by replacing the media with neurobasal culture media containing B27 supplement (Gibco) without antioxidants. After replacement of culture media, they were returned to the normoxic incubator. Control cultures in a solution which is equal to the OGD solution but containing glucose (33 mmol/L; control solution) were maintained in a normoxic incubator for the identical duration as the OGD cultures, and the incubation solution was changed with reperfusion buffer. The cultured cells were then returned back to a normoxic incubator. To investigate the therapeutic efficacy of MSCs, approximately 1 × 10^3^–1 × 10^6^ naïve MSCs and BNDF-eMSCs were added into the upper chamber of an insert with a 1-μm pore (Falcon; Corning Inc., Corning, New York, NY, USA) following OGD and reoxygenation.

### 4.5. In Vitro Cell Viability, Cytotoxicity, and Oxidative Stress Assays

Twenty-four hours after the cortical neurons were incubated with MSCs, the cell counting kit (CCK)-8 (Dojindo, Kumamoto, Japan) assay was conducted according to the manufacturer’s instructions to determine the relative cell proliferation rate (%) of the cortical neurons. Cytotoxicity was determined by lactate dehydrogenase (LDH) release, according to the manufacturer’s instructions (Roche, Mannheim, Germany). Duplicate measurements were performed for each sample. The level of malondialdehyde (MDA), a marker of oxidative stress, was evaluated in duplicate in cell lysates using the OxiSelect TBARS assay kit containing thiobarbituric acid-reactive substances (Cell Biolabs, San Diego, CA, USA), according to the manufacturer’s instructions.

### 4.6. In Vivo Model of Hypoxic Ischemic Brain Injury

To induce HI brain injury, the right common carotid artery was ligated and exposed to 8% oxygen for 2 h in newborn male Sprague–Dawley rats (Orient Co., Seoul, Korea) at postnatal day (P) 7, as described previously [12,13,37]. After confirming the induction of severe HI brain injury involving >50% of the ipsilateral hemisphere by brain magnetic resonance imaging (MRI) (7.0-Tesla, Bruker-Biospin, 8117 Fällanden, Switzerland) within 2 h after modeling, newborn rat pups were treated with hypothermia at a target temperature of 32 °C for 24 h [12,13,37]. Then, the rat pups were randomly allocated into five study groups in a blinded manner as follows: HI control group (n = 29), HI with naïve MSCs (approximately 1 × 10^5^ cells) transplantation group (n = 24); HI with dose 1 BDNF-eMSCs (approximately 1 × 10^4^ cells) transplantation group (n = 26); HI with dose 2 BDNF -eMSCs (approximately 5 × 10^4^ cells) transplantation group (n = 27); and HI with dose 3 BDNF-eMSCs (approximately 1 × 10^5^ cells) transplantation group (n = 28). Varying doses of MSCs in 10 μL saline were administered into the ipsilateral right lateral ventricle using a stereotactic method (Digital Stereotaxic Instrument with fine drive, MyNeurolab, St. Louis, MO, USA; coordinates, x = +0.5, y = +1.2, z = −2.7 mm relative to bregma) in each study group, and an equal volume (10 μL) of saline was administered to the HI control group [12,13,37]. For short-term follow-up study, brain tissues were obtained from approximately half of the animals in each study group for biochemical and histological assessment at the 11th postnatal day (3 days after HI modeling). For long-term follow-up, functional behavioral tests, including a negative geotaxis test and a rotarod test, were performed, and brain MRI was performed to monitor changes in the brain injury volume at P42 (5 weeks after HI induction) in about half of the remaining animals in each study group.

### 4.7. Tissue Preparation and H&E Staining

Whole brain tissues were extracted and weighed 3 days after HI brain injury modeling. For histological analyses, tissues were fixed with 4% formaldehyde solution at room temperature and embedded in paraffin blocks. The tissues were sliced into 4 µm coronal sections, deparaffinized in xylene, and stained for morphological analyses. Four coronal sections were stained with hematoxylin and eosin (H&E) (+1.80 mm to −1.80 mm/bregma).

Brain injury was determined by intact ipsilateral-to-whole-contralateral hemispheric brain area ratio and the brain histopathological scoring system modified by Hedtjärn et al. [38] and Arteaga et al. [39]. The analyses were performed by an investigator blinded to the experimental groups. The intact area was measured using ImageJ software (National Institutes of National Institutes of Health, Bethesda, MD, USA). The brain injury scoring was graded from 0 to 5 in the cortex, striatum, hippocampus, and striatum (with 0 indicating no observable brain atrophy and injury, and 5 indicating severe infarction or tissue loss in most of the hemisphere). The total score, graded from 0 to 20, was the sum of all four regions.

### 4.8. Immunohistochemistry

The microglia activation and reactive gliosis and were histologically assessed by immunohistochemical staining for ED-1 (Ab31630, Abcam, Cambridge, UK) indicating reactive microglial marker and glial fibrillary acidic protein (GFAP) (Z0334, Dako, Glostrup, Denmark) indicating astrocytic glial marker. The number of ED-1-positive cells and light intensity of GFAP were assessed blindly using three non-overlapping fields in three coronal-sectioned brains (+0.95 mm to −0.11 mm/bregma) in the peri-infarct area of each brain.

### 4.9. TUNEL Assay

According to the manufacturer’s protocol, brain cell death was assessed using terminal transferase-mediated biotin dUTP nick end labeling (TUNEL) assay (kit G3250, Promega, Madison, WI, USA). We blindly evaluated the number of TUNEL positive cells using three non-overlapping fields in three coronal sectioned brains (+0.95 mm to −0.11 mm/bregma) in the peri-infarct area of each brain.

### 4.10. Enzyme Linked Immunosorbent Assay

Enzyme-linked immunosorbent assay (ELISA) was performed to confirm expression level of BDNF. Three different passages of BDNF-eMSCs were used for ELISA assay. BDNF-eMSCs were seeded at a density of 1 × 10^5^ cells per 12-wells (Corning) with 1 mL complete media. After 48 h in the 37 °C, 5% CO_2_ incubator, supernatants were harvested and centrifuged to remove cell debris. BDNF concentrations were determined using a Human Free BDNF Quantikine ELISA Kit (R&D Systems, Minneapolis, MN, USA), according to the manufacturer’s instructions. The absorbance of the samples at 450 nm was measured using SpectraMAX 190 (Molecular Devices, San Jose, CA, USA) microplate reader.

BDNF levels were determined in cell culture media using a ProcartaPlex Multiplex ELISA Kit according to the manufacturer’s protocol (eBioscience, Wien, Austria). Brain inflammatory cytokines, including interleukin (IL)-1α, IL-1β, IL-6, and tumor necrosis factor (TNF)-α, were estimated in the peri-infarct area of the brain tissues using a MILLIPLEX MAP ELISA Kit, according to the manufacturer’s protocol (EMD Millipore, Billerica, MA, USA).

### 4.11. Functional Behavioral Tests

The negative geotaxis test was performed at P35 and P42 as previously reported [10,11,13]. Rotarod tests were performed consecutively from P40 to P42, as previously reported [10,11,13]. Behavior function tests were performed and evaluated by investigators blinded to the experimental groups.

### 4.12. Statistical Analysis

Data are expressed as mean ± standard error of the mean (SEM). All data had a significant normal distribution. One-way analysis of variance (ANOVA) and Tukey’s post hoc test were performed to determine the statistical significance between groups for continuous variables. Statistical significance was set at *p* < 0.05. All data were analyzed using SPSS version 18.0 (IBM, Chicago, IL, USA).

## 5. Conclusions

Along with hypersecretion of BDNF, the irradiated BDNF-eMSCs significantly attenuated the OGD-induced cytotoxicity, oxidative stress, and cell death than naïve MSCs in vitro. In the in vivo neonatal HI brain injury animal model, short-term brain injury scores, long-term progress of brain infarct, and impaired behavioral function tests were significantly attenuated only with irradiated BDNF-eMSCs, but not with naïve MSCs transplantation. These findings suggest that MSCs transfected with the BDNF gene might be more useful than naïve MSCs and, thus, represent a better new therapeutic strategy against severe neonatal HI brain injury.

## Data Availability

The datasets generated and analyzed during this current study are available from the corresponding author upon reasonable request.
